# Loop Diuretics Are Associated with Increased Risk of Hospital-Acquired Acute Kidney Injury in Adult Patients: A Retrospective Study

**DOI:** 10.3390/jcm11133665

**Published:** 2022-06-24

**Authors:** Liping Zhou, Yanqin Li, Qi Gao, Yuxin Lin, Licong Su, Ruixuan Chen, Yue Cao, Ruqi Xu, Fan Luo, Peiyan Gao, Xiaodong Zhang, Pingping Li, Sheng Nie, Ying Tang, Xin Xu, on behalf of the EACH Study Investigators

**Affiliations:** 1 National Clinical Research Center for Kidney Disease, State Key Laboratory of Organ Failure Research, Division of Nephrology, Nanfang Hospital of Southern Medical University, Guangzhou 510515, China; 15622157719@163.com (L.Z.); liyanqin819@163.com (Y.L.); qigao_97@163.com (Q.G.); yolo_1997lin@163.com (Y.L.); slc666@smu.edu.cn (L.S.); chenrx114@163.com (R.C.); 4235451815@163.com (Y.C.); xrq_129@163.com (R.X.); 17819566557@163.com (F.L.); gaopeiyan7218@163.com (P.G.); xdzhang_2020@163.com (X.Z.); lpp_smu@163.com (P.L.); niesheng0202@126.com (S.N.); 2 Division of Nephrology, The Third Affiliated Hospital of Southern Medical University, Guangzhou 510515, China

**Keywords:** loop diuretics, acute kidney injury, time-dependent, propensity score match

## Abstract

**Background:** The association between loop diuretics and acute kidney injury (AKI) remains unclear. **Methods:** The population studied was selected from the Epidemiology of AKI in Chinese Hospitalized patients (EACH) study. Exposure to loop diuretics was defined as any filled prescription prior to the date when AKI was detected in patients with HA-AKI, and prior to the last date of SCr testing in those without AKI. The outcome was AKI, defined by the Kidney Disease Improving Global Outcomes criteria. Associations between loop diuretics and HA-AKI were examined by Cox proportional hazards models adjusted for baseline and time-dependent covariates. **Results:** Of the 150,020 patients, 16,437 (11.0%) were prescribed loop diuretics, and 5717 (3.8%) experienced HA-AKI events. The crude rates of HA-AKI in patients who were and were not prescribed loop diuretics were 1632 (9.9%) and 3262 (2.8%), respectively. A multivariate cox proportional hazards analysis showed that exposure to loop diuretics was associated with significantly increased risks of HA-AKI compared with non-users (hazard ratio (HR), 1.61; 95% CI (confidence interval), 1.55–1.67), other diuretics (HR, 1.09; 95% CI, 1.03–1.15), and osmotic diuretics (HR, 1.30; 95% CI, 1.20–1.42). Compared with other diuretics, the use of loop diuretics was associated with higher risks of HA-AKI in women, in patients without hypertension, in patients without heart failure, in patients without liver cirrhosis, and in patients not requiring surgery. **Conclusions:** Loop diuretics are widely used and are associated with increased risks of HA-AKI in hospitalized adults. Renal function should be more closely monitored during the use of loop diuretics.

## 1. Introduction

Hospital-acquired acute kidney injury (HA-AKI) is a rapidly growing challenge for healthcare providers and clinicians worldwide, with an incidence ranging from 11.6% to 18.3% [1,2,3,4,5], affecting over 5000 patients per million individuals, and causing over 1.7 million deaths per year [6,7]. Identifying potentially modifiable factors to reduce the occurrence of AKI is important. Thus far, AKI has been associated with exposure to nephrotoxic medications [3,8,9,10]—especially to widely prescribed and highly effective drugs such as proton-pump inhibitors and diuretics [10].

Diuretics are the most widely prescribed class of fluid management medications indicated for water–sodium retention caused by, for example, hypertension, chronic kidney disease (CKD), or heart failure. Diuretics, which increase urine output, are also the most commonly prescribed class of drugs used to treat AKI [11,12], with loop diuretics being used most frequently in these patients. A case series from Russia in 1995 reported that furosemide treatment induced AKI in 22 patients with glomerulonephritis [13]. Recently, several studies have assessed the relationship between loop diuretics and AKI [14,15,16,17,18]. Most of these studies, however, examined the effects of loop diuretics in critically ill patients, making their effects in general populations unclear [14,15,16,17,18]. Furthermore, AKI in many of these studies was determined from diagnostic codes rather than from serum creatinine (SCr) concentrations [15,17,18], which is a drawback in that diagnostic codes are suboptimal for diagnosing AKI [19]. Additionally, no studies have comprehensively investigated factors that modify the association of loop diuretics with AKI.

The current study aimed to analyze the relationship between treatment with loop diuretics and HA-AKI in hospitalized adults, as well as possible factors modifying this association.

## 2. Materials and Methods

### 2.1. Study Design and Data Source

The study population was selected from the Epidemiology of AKI in Chinese Hospitalized patients (EACH) study—a large, multicenter, retrospective cohort study involving 3,044,244 patients admitted to 25 tertiary public hospitals in 15 provinces throughout China from 2013 to 2015. Patient data were obtained from the electronic medical records system. Age, gender, residence, dates of admission and discharge, diagnosis codes at diagnosis and discharge, surgical procedures and dates, need for admission to the intensive care unit (ICU), values and times of patients’ SCr tests, the name and dosage of each agent, the method and frequency of administration, and starting and stopping times were all collected in the database. The National Clinical Research Center for Kidney Disease in Guangzhou pooled and cleaned the exported data from all study hospitals.

### 2.2. Study Population

**Figure 1:**
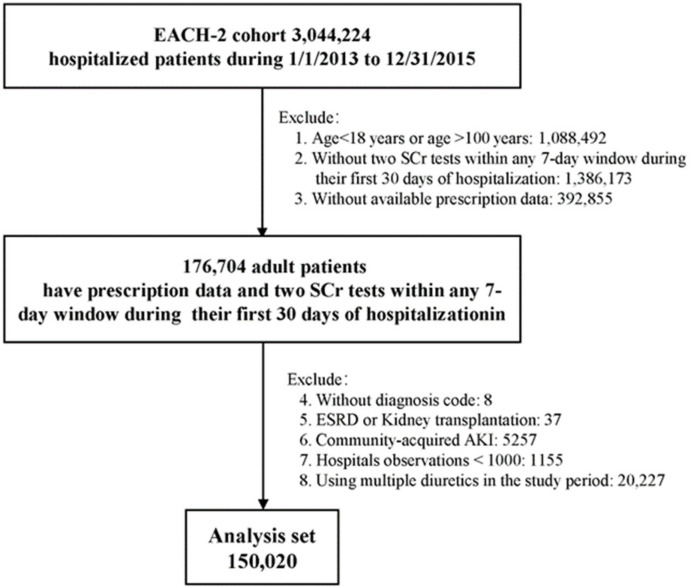
Flowchart of the study population.

The study population included inpatients aged 18 to 100 years admitted between 1 January 2013 and 31 December 2015. Patients were included if prescription data were available and if they underwent two SCr tests within any 7-day window during their first 30 days of hospitalization. Patients were excluded if they had end-stage renal disease, were receiving maintenance dialysis, or were candidates for renal transplantation. Patients were also excluded if they did not have a diagnostic code or were diagnosed with community-acquired (CA) AKI, with the latter defined as a diagnostic code for AKI at admission or a change in SCr on the first day of admission, thus meeting the Kidney Disease Improving Global Outcomes (KDIGO) definition of AKI [3,20,21]. Patients admitted to hospitals with fewer than 1000 individuals or patients treated with multiple diuretics throughout the study period were also excluded (Figure 1). Only the records from the first hospitalization were analyzed for each patient.

The study was conducted in accordance with the Declaration of Helsinki (as revised in 2013). The Medical Ethics Committee of Nanfang Hospital (no. NFEC-2014-098) approved the study, and individual consent for this retrospective analysis was waived.

### 2.3. Identification of HA-AKI

The primary outcome was HA-AKI, defined as a ≥26.5 μmol/L (≥0.3 mg/dL) increase in SCr concentration within 48 h or a ≥50% increase within 7 days, according to the KDIGO criteria [22]. AKI events were screened using a previously described algorithm [3,20,21]. Briefly, at any time point t, baseline SCr was dynamically defined as the mean SCr concentration level within the previous 90 days before time t. Each measured SCr within the 7 days after time *t* was compared with baseline SCr. The earliest day that the change in SCr met the KDIGO criteria was defined as the date of AKI detection.

The stage of AKI was based on the peak SCr after AKI detection, with stages 1, 2, and 3 defined as increases of <100%, 100–199%, and ≥200%, respectively, compared with baseline.

### 2.4. Exposure to Loop Diuretics and Other Medications

Exposure to loop diuretics and other medicines was defined based on any filled prescriptions for these specified drugs prior to the date of detection of AKI in patients with AKI, and prior to the last SCr test in patients without AKI, based on the product codes of the Anatomical Therapeutic Chemical (ATC) Classification System (www.whocc.no/, accessed on 23 October 2019). The cumulative days of drug usage were added up by the difference in days between the starting and stopping time of each period of consecutive drug usage. The cumulative dose of each medication was the product of the daily cumulative dose and the cumulative days of use. The maximum daily dose of a specific medication received by each individual was defined as the maximum of all daily doses for that specific drug.

The prescribed loop diuretics included furosemide and torasemide. Other diuretics included thiazide, low-ceiling (excluding thiazides), potassium-sparing, and osmotic diuretics. Other medications included angiotensin-converting enzyme inhibitors (ACEIs), angiotensin receptor blockers (ARBs), chemotherapy agents, contrast media, glucocorticoids, non-steroidal anti-inflammatory drugs (NSAIDs), proton-pump inhibitors (PPIs), antimycotics, anti-tuberculosis drugs, antiviral drugs, aminoglycosides, first-generation cephalosporins, and semi-synthetic penicillin.

### 2.5. Assessment of Comorbidities and Need for Operation

Relevant comorbid conditions and surgical procedures were recorded based on inpatient diagnoses and procedures, laboratory test results, and drugs prescribed. The presence of comorbidities was identified using International Classification of Diseases, Tenth Revision (ICD-10) diagnosis codes, and the performance of surgical procedures was identified using ICD-9 procedure codes.

### 2.6. Statistical Analysis

Continuous variables were presented as the mean ± standard deviation (SD) or median (25th, 75th quartiles), whereas categorical variables were presented as counts and proportions. Because small differences in large sample sizes can be easily detected using statistical tests, standardized differences in the means or ratios of the study groups were applied to determine differences in patient characteristics; a value of 0.10 was considered a potentially meaningful difference [23].

The effect of loop diuretics on the risk of HA-AKI was estimated using one univariate Cox proportional hazards model and three hierarchical Cox proportional hazards models. Multivariable Model 1 was adjusted for age and gender only, while Model 2 was adjusted for estimated glomerular filtration rate (eGFR) and the number of SCr tests, in addition to the variables in Model 1. Multivariable Model 3 was adjusted for the variables in Model 2, along with the Charlson Comorbidity Index (CCI) score [24], need for admission to the ICU, mechanical ventilation, sepsis, hypertension, diabetes, primary nephritis, CKD, nephrotic syndrome, brain injury, heart failure, hepatitis, liver cirrhosis, gastrointestinal bleeding (GIB), cerebrovascular disease (CVD), systemic lupus erythematosus (SLE), congenital heart disease, urinary tract infection, congenital urinary system malformation (CUSM), respiratory infection, chronic obstructive pulmonary disease (COPD), diarrhea/vomiting, stroke, shock, trauma, burns, coronary heart disease (CHD), malignant solid tumor, hematological malignancy, hepatic carcinoma, gastrointestinal operation, cardiac respiratory operation, neurosurgical operation, orthopedic operation, urinary system operation, interventional operation, ACEIs, ARBs, chemotherapy agents, contrast media, glucocorticoids, non-steroidal anti-inflammatory drugs (NSAIDs), PPIs, antimycotics, anti-tuberculosis drugs, antiviral drugs, aminoglycosides, sulfonamides, first-generation cephalosporins, and semi-synthetic penicillin, and stratified by hospital and division. In addition to loop diuretics as the primary exposure, eGFR, surgical procedures, and other medications were coded as time-dependent variables given these confounders, as they may influence the receipt and persistent use of loop diuretics or the risk of AKI over time.

Possible interactions of loop diuretics with age (18–40, 40–65 or ≥65 years), gender, primary nephritis, nephrotic syndrome, hypertension, heart failure, liver cirrhosis, ICU, operation, and ACEI/ARB were also examined using adjusted Model 3.

A penalized smoothing spline method (degree = 4) was used to test for linearity and explore the shape of the relationships of cumulative dose, maximum daily dose, and cumulative prescription duration of furosemide with HA-AKI.

### 2.7. Sensitivity Analyses

One-to-one propensity score methods matched patients receiving loop diuretics with three other groups: those not receiving diuretics, patients treated with other diuretics, and patients treated with osmotic diuretics. Propensity scores were estimated using a logistic regression model involving covariates of Model 3. Three different matching methods were used to select the control group. Method 1 consisted of nearest-neighbor matching without replacement and within a specified caliper width of 0.001; Method 2 consisted of nearest-neighbor matching without replacement and within a specified caliper width of 0.2 times the SD of the logit of the estimated propensity score; Method 3 consisted of exact matching of age, sex, ICU, surgery, and nearest-neighbor matching without replacement and within a specified caliper width of 0.2 times the SD of the logit of the estimated propensity score on other covariates.

All statistical analyses were performed using R version 3.5.3 for Windows (http://www.Rproject.or-g/, accessed on 11 March 2019).

## 3. Results

### 3.1. Study Population and Baseline Characteristics

A total of 150,020 patients, with a median age of 53.0 years, met our inclusion and exclusion criteria, and were selected for analyses (Figure 1). Of these, 16,437 (11.0%) patients were treated with loop diuretics. The proportions of men and elderly patients were higher in users of loop diuretics than in those not treated with diuretics (Table 1), with both men and elderly patients tending to be exposed to more types of drugs, and being more likely to have complex comorbidities, frequent SCr tests, long hospital stays, ICU admission, surgical procedures, and in-hospital death. Moreover, their baseline SCr was much higher. Of the patients prescribed loop diuretics, 94.2% were prescribed furosemide (Supplementary Table S1). The baseline characteristics of the excluded patients were similar to those included in the final analyses (Supplementary Table S2).

### 3.2. Association between Loop Diuretic Treatment and HA-AKI

An analysis of HA-AKI events showed that a total of 1632 (9.9%), 3262 (2.8%), 823 (4.4%), and 714 (4.2%) occurred in users of loop diuretics, non-users of diuretics, users of other diuretics, and users of osmotic diuretics, respectively. Cox regression analysis showed that loop diuretics were associated with an overall increased risk of HA-AKI. Users of loop diuretics had a 61% higher risk of HA-AKI than non-users of diuretics (hazard ratio (HR) = 1.61; 95% confidence interval (CI), 1.55–1.67) in the adjusted Multivariable Model 3 (Table 2). Moreover, compared with other diuretics that have more similar indications to loop diuretics than non-users, users of loop diuretics had a 9% higher risk of HA-AKI (HR, 1.09; 95% CI, 1.03–1.15). Of the patients using other diuretics, 91.4% were treated with osmotic diuretics (Supplementary Table S1); compared with this group, users of loop diuretics had a 30% higher risk of HA-AKI (HR, 1.30; 95% CI, 1.20–1.42) after adjusting for covariates.

Consistently, treatment with furosemide significantly increased the risk of HA-AKI compared with treatment with other diuretics, osmotic diuretics, and no treatment. Compared with patients treated with torasemide, treatment with furosemide was associated with a 33% higher risk of HA-AKI (HR, 1.33; 95% CI, 1.14–1.54) (Supplementary Table S3).

### 3.3. Potential Modifiers of the Effects of Loop Diuretics on HA-AKI

Multivariable Model 3 with interactions was utilized to further assess the effect of the heterogeneity of loop diuretics on HA-AKI in the setting of various patient characteristics. Compared with other diuretics, the use of loop diuretics was associated with higher risks of HA-AKI in women (*p* for interaction < 0.001), in patients without hypertension (*p* for interaction < 0.001), in patients without heart failure (*p* for interaction < 0.001), in patients without liver cirrhosis (*p* for interaction = 0.02), and in patients not requiring surgery (*p* = 0.02, Table 3). Age, primary nephritis, nephrotic syndrome, and concomitant use of ACEIs/ARBs did not modify the effect of treatment with loop diuretics on HA-AKI. Similar associations were observed between users of loop diuretics and non-users of diuretics (Supplementary Table S4).

### 3.4. Response Curves of Loop Diuretics and HA-AKI

**Figure 2:**
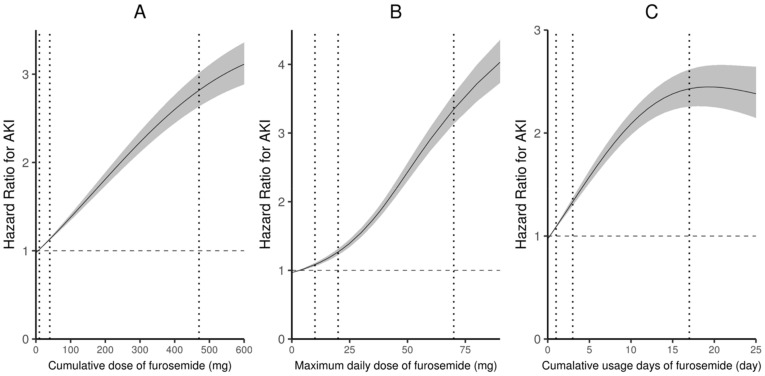
Response curves of furosemide with the risk of HA-AKI based on a penalized smoothing spline. There was a linear, positive association between cumulative dose (**A**), maximum daily dose (**B**), cumulative usage days (**C**), and HA-AKI. Adjusted for age, gender, estimated glomerular filtration rate, number of serum creatinine tests, Charlson Comorbidity Index score, need for admission to the intensive care unit, mechanical ventilation, comorbidities, surgical procedures, and use of other nephrotoxic drugs, and stratified by hospitals and divisions. The leftmost, middle, and rightmost vertical lines indicate 25%, 50%, and 75% of the *x*-axis, respectively. HA-AKI, hospital-acquired acute kidney injury; mg, milligram.

Because 94.2% of the patients prescribed loop diuretics were prescribed furosemide, we hypothesized that the response curves of the relationships between furosemide and HA-AKI were representative of the relationships between loop diuretics and HA-AKI (Figure 2). The dose–hazard ratio curves suggested that there were linear, positive associations between the cumulative dose, maximum daily dose, and cumulative days of treatment with furosemide, and HA-AKI.

### 3.5. Sensitivity Analysis

The distribution of the standardized differences before and after propensity matching of patients treated with loop diuretics with the three other groups of patients (i.e., those not treated with diuretics, patients treated with other diuretics, and patients treated with osmotic diuretics) by the three different matching methods are depicted in Supplementary Figures S1–S3. Method 1, yielding the best balance of post-matched participants, generated 14,490, 3655, and 2272 analytical pairs of patients for comparisons of loop-diuretic-treated patients with the non-diuretic, other diuretics, and osmotic diuretics groups, respectively. Following 1:1 propensity matching by Method 1, treatment with loop diuretics was associated with significantly increased risks of HA-AKI compared to untreated patients (HR, 1.52; 95% CI, 1.45–1.59) and patients treated with other diuretics (HR, 1.21; 95% CI, 1.10–1.34) and osmotic diuretics (HR, 1.14; 95% CI, 1.02–1.27) (Supplementary Table S5). These associations were comparable to those in the main analysis (Table 2), and were consistent across the other two propensity-matching methods (Supplementary Tables S6 and S7). The effect size of this sensitivity analysis was similar to that of the main analysis.

## 4. Discussion

To our knowledge, this is the first large cohort study to focus on the association between the use of loop diuretics and HA-AKI in adults in China. This relatively large, multicenter, retrospective cohort found that 16,437 (11.0%) individuals were prescribed loop diuretics, among whom 9.9% developed HA-AKI. Treatment with loop diuretics was significantly associated with the development of HA-AKI in hospitalized adults. Factors associated with susceptibility to HA-AKI included female sex, the absence of hypertension, the absence of heart failure, the absence of liver cirrhosis, and the lack of a need for surgery.

To date, various studies have explored whether loop diuretics are associated with an increased risk of AKI. For example, a study of 132 critically ill patients suggested that the use of furosemide was associated with the development of AKI in patients with sepsis/septic shock [14]. In addition, a nested case–control study that included 487,372 patients reported that the combination of diuretics with ACEIs/ARBs and NSAIDs was associated with an increased risk of AKI [15]. Meanwhile, another nested case–control study comprising 78,379 patients found that diuretics synergistically amplified the adverse renal effects of nephrotoxic drugs such as NSAIDs and renin–angiotensin system inhibitors (RASIs) [16]. Moreover, a population-based cohort study that included 140,952 individuals concluded that exposure to RAAS inhibitors or diuretics increased the risk of AKI [17], and a retrospective cohort study of 252 patients found that an initially high dose of intravenous loop diuretics was associated with an increased risk of developing AKI [18]. These studies, however, included relatively few or relatively high-risk patients, and several did not explore the relationship between loop diuretics and AKI separately, obscuring the ability to accurately assess the relationship between loop diuretics and HA-AKI. However, the ability to evaluate these relationships could be improved by using a more reasonable definition of AKI, selecting a more representative population, and adjusting for more meaningful confounders.

In our study, the use of a more rigorous analytic approach and the inclusion of baseline and potential time-dependent confounders may provide some greater insights into the association between loop diuretics and HA-AKI. First, this comparison remained significant, regardless of whether the patients treated with loop diuretics were compared with groups of patients who were not treated with diuretics or who were treated with osmotic or other diuretics—a finding consistent with the results of previous studies [14,15,16,17,18]. As with any large observational study, the risk of confounding by indication is large, and cannot be ignored. However, the robustness of our results is additionally bolstered by the strengths of several analytical methods, including accessibility to patients’ SCr data, including both concentrations and times of measurement stamps. Patients were included if they had undergone two SCr tests within any 7-day window during their first 30 days of hospitalization [3]. Our algorithm allowed us to determine a higher risk of HA-AKI among loop diuretics users as a more accurate reflection of HA-AKI under real-world conditions. In addition, we encoded eGFR, all surgical procedures, and all medications relevant to the initiation of loop diuretic therapy and AKI status, and modeled them as potential time-dependent confounders using Cox proportional hazards models. Moreover, users of loop diuretics were compared not only with non-users of diuretics, but also with users of osmotic diuretics and other diuretics as active controls, as the indications for osmotic and other diuretics may be similar to the indications for loop diuretics. We also utilized three different propensity-score-matching methods to ensure that the post-matched participants would be most closely balanced for sensitivity analyses. Finally, the response curves showed the shape of the relationship between loop diuretics and HA-AKI.

Second, although the biological mechanisms underlying the association between loop diuretics and increased risk of AKI remain to be elucidated, our findings are biologically plausible. Reduced kidney perfusion is regarded as a major modifiable risk factor for AKI [25]. Prerenal factors reduce effective circulating blood volume, leading to insufficient renal perfusion, which can cause AKI [26,27]. Loop diuretics inhibit the reabsorption of sodium in the thick ascending limbs of Henle, which can lead to an improvement in filling pressure and relief of water load. Due to various causes, however—such as activation of the renin–angiotensin system or the sympathetic nervous system—treatment with loop diuretics may also result in volume depletion and possible renal hypoperfusion, further reducing kidney function [28]. Furosemide has also been found to result in a relative decrease in medullary blood flow when compared with the cortex [29,30,31]. Additional studies are warranted to further examine the mechanisms underlying the induction of AKI by loop diuretics.

As we know, loop diuretics are mostly used to control volume in hospitalized patients—especially in patients with congestive heart failure (CHF) and fluid overload. After patients with CHF use loop diuretics, a slight increase in creatinine levels is called “permissive hypercreatininemia”. However, this change in creatinine levels is usually a slight increase, rather than a short-term increase of over 50%. Our analysis mainly focused on significant acute increases in serum creatinine, which reached the diagnostic level of AKI. We suggest that renal function should be more closely monitored during the use of diuretics, and that clinicians should pay more attention to the possible occurrence of AKI after using loop diuretics.

Another point worth mentioning in this research was that gender, hypertension, heart failure, liver cirrhosis, and surgery may modify the association between loop diuretic and HA-AKI, with a higher risk in women, patients without hypertension, patients without heart failure, patients without liver cirrhosis, and those not requiring surgery. In contrast, previous studies of hospitalized patients showed that the frequency of AKI was higher in older than in younger patients, in men than in women, and in patients with than without comorbidities such as diabetes, hypertension, heart failure, and surgery [3,32,33]. We speculate that the use of loop diuretics in patients with hypertension or requiring surgery may be associated with a lower risk of HA-AKI because clinicians may pay more attention to their fluid balance, and are more cautious when prescribing diuretics, with their benefits outweighing adverse renal events. In addition, loop diuretics are rarely used to treat hypertension, unless complicated by heart failure, which is the most common indication for diuretic use. Liver cirrhosis—especially in patients with cirrhosis and portal hypertension with ascites and generalized anasarca—is also among the most common indications for diuretic use. Our study found that heart failure and liver cirrhosis may modify the association between loop diuretic use and HA-AKI, perhaps because of these two conditions being indications for diuretic use. Further studies are needed to verify this hypothesis.

This study had several limitations. First, AKI was not diagnosed by measuring urine output, because urine output was generally not measured in the general hospital wards. Second, the study cohort only included Chinese adults, which suggests the need for further validation to determine whether these results could be generalized to other ethnic populations. Third, despite our attempts to adjust for potential confounders using both a traditional approach and after propensity score-matching, there remains—as in other observational studies—a risk of residual confounding that could not be controlled. Additional studies are required to confirm these results. Fourth, the high proportion of missing prescription information before admission made it impossible to determine whether patients were chronically treated with loop diuretics, started treatment with diuretics for an acute condition when hospitalized, or both. Finally, we note that the observed associations do not imply causation.

## 5. Conclusions

In summary, the results of this study suggest that treatment with loop diuretics is common in hospitalized Chinese adults, and is associated with increased risks of HA-AKI. These findings suggest the need to balance the advantages and disadvantages of loop diuretics in individual patients, as well as the need for more definitive evidence from randomized, controlled trials.

## Figures and Tables

**Table 1:** Characteristics of hospitalized adults stratified by use of diuretics.

Characteristic	All	Non-Users	Loop Diuretics	Other Diuretics *	Sd
N (%)	150,020	114,815 (76.5)	16,437 (11.0)	18,768 (12.5)	
Age, years	53.0 [41.8, 63.8]	52.4 [41.0, 63.5]	58.7 [47.1, 67.9]	51.9 [42.2, 61.3]	0.26
Male	79,638 (53.1)	58,406 (50.9)	10,284 (62.6)	10,948 (58.3)	0.16
Baseline SCr, μmol/L	60.8 [50.4, 74.0]	60.5 [50.5, 73.8]	65.9 [53.4, 82.5]	57.8 [48.0, 69.0]	0.28
Baseline eGFR, mL/min/1.73 m^2^	103.4 [91.4, 115.3]	103.5 [91.7, 115.4]	97.3 [80.9, 109.4]	107.5 [97.5, 118.2]	0.40
Number of SCr tests	2.4 ± 2.5	2.0 ± 1.9	4.2 ± 4.1	2.9 ± 3.3	0.47
AKI	5717 (3.8)	3262 (2.8)	1632 (9.9)	823 (4.4)	0.20
AKI stage					0.20
1	4004 (2.7)	2366 (2.1)	1124 (6.8)	514 (2.7)	
2	973 (0.6)	512 (0.4)	286 (1.7)	175 (0.9)	
3	740 (0.5)	384 (0.3)	222 (1.4)	134 (0.7)	
Length of stay, days	13.0 [9.0, 20.0]	12.0 [8.0, 18.0]	19.0 [12.0, 27.0]	16.0 [12.0, 23.0]	0.31
Daily cost, CNY	2255 [1149, 3575]	2103 [1077, 3365]	2715 [1565, 3870]	2920 [1527, 4547]	0.22
In-hospital death	852 (0.6)	327 (0.3)	314 (1.9)	211 (1.1)	0.11
CCI	3.0 [2.0, 4.0]	3.0 [2.0, 4.0]	4.0 [2.0, 5.0]	3.0 [2.0, 4.0]	0.29
ICU	9358 (6.2)	5216 (4.5)	1404 (8.5)	2738 (14.6)	0.23
Mechanical ventilation	212 (0.1)	66 (0.1)	63 (0.4)	83 (0.4)	0.05
**Comorbidity**					
Hypertension	8201 (5.5)	6562 (5.7)	941 (5.7)	698 (3.7)	0.06
Heart failure	923 (0.6)	507 (0.4)	352 (2.1)	64 (0.3)	0.11
Congenital heart disease	360 (0.2)	250 (0.2)	60 (0.4)	50 (0.3)	0.02
CHD	5787 (3.9)	4748 (4.1)	727 (4.4)	312 (1.7)	0.10
Cerebral disease	3886 (2.6)	1289 (1.1)	126 (0.8)	2471 (13.2)	0.34
Stroke	7715 (5.1)	3352 (2.9)	467 (2.8)	3896 (20.8)	0.39
CVD	8783 (5.9)	3834 (3.3)	520 (3.2)	4429 (23.6)	0.42
Primary nephritis	936 (0.6)	790 (0.7)	119 (0.7)	27 (0.1)	0.06
CKD	3906 (2.6)	2948 (2.6)	797 (4.8)	161 (0.9)	0.17
Nephrotic syndrome	1745 (1.2)	1309 (1.1)	367 (2.2)	69 (0.4)	0.11
Urinary tract infection	1195 (0.8)	938 (0.8)	147 (0.9)	110 (0.6)	0.02
CUSM	245 (0.2)	202 (0.2)	36 (0.2)	7 (0.0)	0.03
Diabetes	4249 (2.8)	3188 (2.8)	468 (2.8)	593 (3.2)	0.02
SLE	1,045 (0.7)	859 (0.7)	144 (0.9)	42 (0.2)	0.06
Hepatitis	5410 (3.6)	4250 (3.7)	904 (5.5)	256 (1.4)	0.16
Liver cirrhosis	2223 (1.5)	1439 (1.3)	693 (4.2)	91 (0.5)	0.17
GIB	1629 (1.1)	1239 (1.1)	292 (1.8)	98 (0.5)	0.08
Respiratory infection	9382 (6.3)	6421 (5.6)	1673 (10.2)	1288 (6.9)	0.11
COPD	2459 (1.6)	1807 (1.6)	546 (3.3)	106 (0.6)	0.14
Sepsis	598 (0.4)	412 (0.4)	147 (0.9)	39 (0.2)	0.06
Shock	734 (0.5)	399 (0.3)	227 (1.4)	108 (0.6)	0.08
Trauma	6598 (4.4)	3200 (2.8)	408 (2.5)	2990 (15.9)	0.32
Burns	512 (0.3)	327 (0.3)	124 (0.8)	61 (0.3)	0.04
Malignant solid tumor	39,370 (26.2)	29,997 (26.1)	6960 (42.3)	2413 (12.9)	0.46
Hematological malignancy	3737 (2.5)	2914 (2.5)	675 (4.1)	148 (0.8)	0.15
Hepatic carcinoma	5101 (3.4)	3798 (3.3)	1210 (7.4)	93 (0.5)	0.25
**Receiving operation**	85,598 (57.1)	64,196 (55.9)	9747 (59.3)	11655 (62.1)	0.08
Gastrointestinal operation	18,502 (12.3)	14,313 (12.5)	3974 (24.2)	215 (1.1)	0.50
Cardiac respiratory operation	8321 (5.5)	5450 (4.7)	2164 (13.2)	707 (3.8)	0.23
Neurosurgical operation	7600 (5.1)	2247 (2.0)	60 (0.4)	5293 (28.2)	0.60
Orthopedic operation	20,936 (14)	16,066 (14.0)	1193 (7.3)	3677 (19.6)	0.25
Urinary system operation	5114 (3.4)	4099 (3.6)	948 (5.8)	67 (0.4)	0.22
Interventional operation	7829 (5.2)	5525 (4.8)	643 (3.9)	1661 (8.9)	0.14
**Medication use**					
ACEI	5013 (3.3)	3488 (3.0)	823 (5.0)	702 (3.7)	0.07
ARB	9513 (6.3)	7067 (6.2)	1044 (6.4)	1402 (7.5)	0.04
Chemotherapy agents	22,167 (14.8)	17,625 (15.4)	3187 (19.4)	1355 (7.2)	0.24
Contrast media	1185 (0.8)	814 (0.7)	138 (0.8)	233 (1.2)	0.04
Glucocorticoids	39,433 (26.3)	24,211 (21.1)	6979 (42.5)	8243 (43.9)	0.34
NSAIDs	67,089 (44.7)	49,997 (43.5)	8403 (51.1)	8689 (46.3)	0.10
PPI	114,385 (76.2)	85,265 (74.3)	13,536 (82.4)	15,584 (83.0)	0.14
Antimycotics	4219 (2.8)	2438 (2.1)	1398 (8.5)	383 (2.0)	0.20
Anti-tuberculosis drugs	2002 (1.3)	1462 (1.3)	281 (1.7)	259 (1.4)	0.02
Antiviral drugs	6457 (4.3)	4578 (4.0)	1243 (7.6)	636 (3.4)	0.12
Aminoglycosides	4754 (3.2)	2910 (2.5)	1294 (7.9)	550 (2.9)	0.16
Sulfonamides	609 (0.4)	307 (0.3)	214 (1.3)	88 (0.5)	0.08
First-generation cephalosporins	1890 (1.3)	1300 (1.1)	336 (2.0)	254 (1.4)	0.05
Semi-synthetic penicillin	34,843 (23.2)	23,774 (20.7)	5962 (36.3)	5107 (27.2)	0.23

Note: the number of SCr tests is presented in mean ± SD; values expressed with numbers and square brackets are presented as median [25th, 75th quartiles]; values expressed with numbers and round brackets are presented as count (percentage). * Other diuretics include thiazide, low-ceiling (excluding thiazides), potassium-sparing, and osmotic diuretics. Abbreviations: Sd, standardized difference; SCr, serum creatinine; eGFR, estimated glomerular filtration rate; AKI, acute kidney injury; CNY, Chinese Yuan; CCI, Charlson Comorbidity Index score; ICU, need for admission to the intensive care unit; CHD, coronary heart disease; CVD, cardiovascular disease; CKD, chronic kidney disease; CUSM, congenital urinary system malformation; SLE, systemic lupus erythematosus; GIB, gastrointestinal bleeding; COPD, chronic obstructive pulmonary disease; ACEI, angiotensin-converting enzyme inhibitor; ARB, angiotensin receptor blocker; NSAIDs, non-steroidal anti-inflammatory drugs; PPI, proton-pump inhibitor.

**Table 2:** The association between treatment with diuretics and the risk of HA-AKI.

Subgroup	HA-AKI/N, (%)	Crude Model		Adjusted Model 1		Adjusted Model 2		Adjusted Model 3	
		HR (95% CI)	*p*-Value	HR (95% CI)	*p* Value	(95% CI)	*p*-Value	HR (95% CI)	*p*-Value
**Loop Diuretics Versus Non-Users**									
Non-users	3262/11,4815 (2.8)	Ref.		Ref.		Ref.		Ref.	
Loop diuretics	1632/16,437 (9.9)	3.06 (2.96, 3.17)	<0.001	2.83 (2.73, 2.93)	<0.001	2.13 (2.06, 2.20)	<0.001	1.61 (1.55, 1.67)	<0.001
Loop Diuretics Versus Other Diuretics *									
Other diuretics	823/18,768 (4.4)	Ref.		Ref.		Ref.		Ref.	
Loop diuretics	1632/16,437 (9.9)	2.04 (1.95, 2.13)	<0.001	1.88 (1.80, 1.96)	<0.001	1.48 (1.42, 1.55)	<0.001	1.09 (1.03, 1.15)	<0.001
**Loop Diuretics Versus Osmotic Diuretics**									
Osmotic diuretics	714/17,151 (4.2)	Ref.		Ref.		Ref.		Ref.	
Loop diuretics	1632/16,437 (9.9)	2.50 (2.38, 2.62)	<0.001	2.35 (2.24, 2.47)	<0.001	1.64 (1.56, 1.73)	<0.001	1.30 (1.20, 1.42)	<0.001

Abbreviations: HA-AKI, hospital-acquired acute kidney injury; HR, hazard ratio; CI, confidence interval; Ref, reference. * Other diuretics include thiazide, low-ceiling (excluding thiazides), potassium-sparing, and osmotic diuretics. Model 1, adjusted for age and gender; Model 2, adjusted for estimated glomerular filtration rate (eGFR) and number of SCr tests, in addition to the variables in Model 1; Model 3, adjusted for the variables in Model 2, along with the Charlson Comorbidity Index score, need for admission to the intensive care unit, mechanical ventilation, comorbidities, surgical procedures, and use of other nephrotoxic drugs, and stratified by hospitals and divisions.

**Table 3:** The association between loop diuretics versus other diuretics and the risk of HA-AKI in various subgroups.

Subgroup	Loop Diuretics	Other Diuretics *	Adjusted HR ^#^(95% CI)	*p* for Interaction
	HA-AKI/Total (%)	HA-AKI/Total (%)		
Age				0.10
18–40 years	192/2332 (8.2)	155/3948 (3.9)	1.22 (1.09, 1.37)	
40–65 years	815/8815 (9.2)	496/11,670 (4.3)	1.29 (1.19, 1.40)	
65–100 years	625/5290 (11.8)	172/3150 (5.5)	1.38 (1.25, 1.52)	
Gender				<0.001
Male	1089/10,284 (10.6)	542/10,948 (5.0)	1.22 (1.13, 1.32)	
Female	543/6153 (8.8)	281/7820 (3.6)	1.51 (1.37, 1.67)	
Primary nephritis				0.35
Yes	12/119 (10.1)	1/27 (3.7)	3.09 (0.42, 22.82)	
No	1620/16,318 (9.9)	822/18,741 (4.4)	1.30 (1.21, 1.40)	
Nephrotic syndrome				0.17
Yes	46/367 (12.5)	5/69 (7.2)	0.91 (0.55, 1.51)	
No	1586/16,070 (9.9)	818/18,699 (4.4)	1.31 (1.22, 1.41)	
Hypertension				<0.001
Yes	99/941 (10.5)	39/698 (5.6)	0.86 (0.70, 1.06)	
No	1533/15,496 (9.9)	784/18,070 (4.3)	1.34 (1.25, 1.45)	
Heart failure				<0.001
Yes	87/352 (24.7)	17/64 (26.6)	0.48 (0.35, 0.64)	
No	1545/16,085 (9.6)	806/18,704 (4.3)	1.34 (1.24, 1.44)	
Liver cirrhosis				0.02
Yes	50/693 (7.2)	9/91 (9.9)	0.80 (0.54, 1.18)	
No	1582/15,744 (10.0)	814/18,677 (4.4)	1.31 (1.22, 1.41)	
ICU				0.66
Yes	242/1404 (17.2)	223/2738 (8.1)	1.06 (0.94, 1.18)	
No	1490/15,033 (9.9)	600/16,030 (3.7)	1.45 (1.33, 1.58)	
Operation				0.02
Yes	801/9747 (8.2)	386/11,655 (3.3)	1.17 (1.05,1.31)	
No	831/6690 (12.4)	437/7113 (6.1)	1.36 (1.26,1.48)	
ACEI/ARB				0.15
Yes	200/1756 (11.4)	94/1963 (4.8)	1.18 (1.02, 1.37)	
No	1432/14,681 (9.7)	729/16,805 (4.3)	1.33 (1.24, 1.44)	

Abbreviations: HA-AKI, hospital-acquired acute kidney injury; HR, hazard ratio; CI, confidence interval; ICU, need for admission to the intensive care unit; ACEI, angiotensin-converting enzyme inhibitor; ARB, angiotensin receptor blocker. * Other diuretics include thiazide, low-ceiling (excluding thiazides), potassium-sparing, and osmotic diuretics. # Adjusted for age, gender, estimated glomerular filtration rate, number of serum creatinine tests, Charlson Comorbidity Index score, need for admission to the intensive care unit, mechanical ventilation, comorbidities, surgical procedures, and use of other nephrotoxic drugs, and stratified by hospitals and divisions.

## Data Availability

The datasets generated for this study are available upon request to the corresponding author.

## Supplementary Materials

The following supporting information can be downloaded at: https://www.mdpi.com/article/10.3390/jcm11133665/s1, Table S1. Proportion of diuretics subtypes. Table S2. Characteristics of the included and the excluded adults with prescription data. Table S3. The association between treatment with furosemide and risk of HA-AKI. Table S4. The association between loop diuretics versus non-users and risk of HA-AKI in various subgroups. Table S5. The association between loop diuretics versus non-users and risk of HA-AKI in the 1:1 propensity score-matched cohorts (Method 1). Table S6. The association between loop diuretics versus other diuretics and risk of HA-AKI in the 1:1 propensity score-matched cohorts (Method 2). Table S7. The association between loop diuretics versus osmotic diuretics and risk of HA-AKI in the 1:1 propensity score-matched cohorts (Method 3). Figure S1. Distribution of standardized differences before and after 1:1 propensity score matching by three different models between loop diuretics and non-users. Figure S2. Distribution of standardized differences before and after 1:1 propensity score matching by three different models between loop diuretics and other diuretics. Figure S3. Distribution of standardized differences before and after 1:1 propensity score matching by three different models between loop diuretics and osmotic diuretics.
