# Supplementary material for: Loop Diuretics Are Associated with Increased Risk of Hospital-Acquired Acute Kidney Injury in Adult Patients: A Retrospective Study
Source: J Clin Med. 2022 Jun 24;11(13):3665. doi: 10.3390/jcm11133665 (PMC9267783; doi:10.3390/jcm11133665)
Supplement: Supplementary file 1 [file jcm-11-03665-s001.zip › jcm-1742449-supplementary.pdf]

Supplementary Material

Table S1. Proportion of diuretics subtypes.

Subgroup	ATC code	N (%)
Diuretics	C03/B05BC	35,223 (23.5)
**Loop diuretics**	C03CA	16,437 (11.0)
Furosemide	C03CA01	15,489 (94.2)
Torasemide	C03CA04	948 (5.8)
**Other diuretics***	C03AA/C03BA/C03DA/C03DB/B05BC	18,768 (12.5)
Thiazides	C03AA	243 (0.2)
Hydrochlorothiazide	C03AA03	243 (100)
Low-ceiling diuretics (excluding thiazides)	C03B	486 (0.3)
indapamide	C03BA11	486 (100)
Potassium-sparing diuretics	C03D	888 (0.6)
spironolactone	C03DA01	781 (88.0)
amiloride	C03DB01	110 (12.4)
osmotic diuretics	B05BC	17,151 (11.4)
mannitol	B05BC01	17,103 (99.7)
carbamide	B05BC02	51 (0.3)

Abbreviation: ATC, Anatomical Therapeutic Chemical classification.

*Other diuretics include thiazide, Low-ceiling (excluding thiazides), potassium-sparing and osmotic diuretics.

Table S2. Characteristics of the included and the excluded adults with prescription data.

Characteristic	Included adults	Excluded adults with prescription data	Standardized Difference
N	150,020	254,491	
Age, year	53.0 [41.9, 63.8]	51.4 [37.4, 63.2]	0.11
Male, n (%)	79,638 (53.1)	128,461 (50.5)	0.05
Number of SCr tests	2.4±2.5	1.3±1.6	0.53
Length of stay, day	13.0 [9.0, 20.0]	8.0 [5.0, 11.0]	0.60
Daily cost, CNY	2,255 [1149, 3575]	1,257 [812, 2159]	0.30
In-hospital death, n (%)	852 (0.6)	529 (0.2)	0.06
CCI	3.0 [2.0, 4.0]	3.0 [1.0, 4.0]	0.16
ICU, n (%)	9,358 (6.2)	9,892 (3.9)	0.11
Mechanical ventilation, n (%)	212 (0.1)	102 (0.0)	0.03
Comorbidity, n (%)			
Hypertension	8,201 (5.5)	18,668 (7.3)	0.08
Heart failure	923 (0.6)	3,148 (1.2)	0.06
Congenital heart disease	360 (0.2)	940 (0.4)	0.02
CHD	5,787 (3.9)	14,113 (5.5)	0.08
Cerebral disease	3,886 (2.6)	2,802 (1.1)	0.11
Stroke	7,715 (5.1)	11,086 (4.4)	0.04
CVD	8,783 (5.9)	12,841 (5.0)	0.04
Primary nephritis	936 (0.6)	2,214 (0.9)	0.03
CKD	3,906 (2.6)	6,127 (2.4)	0.01
Nephrotic syndrome	1,745 (1.2)	3,601 (1.4)	0.02
Urinary tract infection	1,195 (0.8)	1,333 (0.5)	0.03
CUSM	245 (0.2)	161 (0.1)	0.03
Diabetes	4,249 (2.8)	6,771 (2.7)	0.01
SLE	1,045 (0.7)	1,419 (0.6)	0.02
Hepatitis	5,410 (3.6)	5,414 (2.1)	0.09
Liver cirrhosis	2,223 (1.5)	2,513 (1.0)	0.05
GIB	1,629 (1.1)	1,945 (0.8)	0.03
Respiratory infection	9,382 (6.3)	11,177 (4.4)	0.08
COPD	2,459 (1.6)	3,765 (1.5)	0.01
Diarrhea/vomiting	273 (0.2)	611 (0.2)	0.01
Sepsis	598 (0.4)	427 (0.2)	0.04
Shock	734 (0.5)	543 (0.2)	0.05
Trauma	6,598 (4.4)	7,614 (3.0)	0.08
Burn	512 (0.3)	853 (0.3)	0.00
Malignant solid tumor	39,370 (26.2)	47,266 (18.6)	0.19
Hematological malignancy	3,737 (2.5)	3,151 (1.2)	0.09
Hepatic carcinoma	5,101 (3.4)	4,976 (2.0)	0.09
Receiving operation, n (%)	85,598 (57.1)	95,927 (37.7)	0.40

Gastrointestinal operation	18,502 (12.3)	8,634 (3.4)	0.34
Cardiac respiratory operation	8,321 (5.5)	7,134 (2.8)	0.14
Neurosurgical operation	7,600 (5.1)	2,508 (1.0)	0.24
Orthopedic operation	20,936 (14.0)	17,625 (6.9)	0.23
Urinary system operation	5,114 (3.4)	2,411 (0.9)	0.17
Interventional operation	7,829 (5.2)	18,793 (7.4)	0.09
Medication use, n (%)			
ACEI	5,013 (3.3)	12,013 (4.7)	0.07
ARB	9,513 (6.3)	24,190 (9.5)	0.12
Chemotherapy agents	22,167 (14.8)	33,897 (13.3)	0.04
Contrast medium	1,185 (0.8)	652 (0.3)	0.07
Glucocorticoid	39,433 (26.3)	53,872 (21.2)	0.12
NSAIDs	67,089 (44.7)	86,209 (33.9)	0.22
PPI	114,385 (76.2)	123,865 (48.7)	0.59
Antimycotics	4,219 (2.8)	2,044 (0.8)	0.15
Anti-tuberculosis drugs	2,002 (1.3)	1,036 (0.4)	0.10
Antiviral drugs	6,457 (4.3)	7,174 (2.8)	0.08
Aminoglycoside	4,754 (3.2)	2,691 (1.1)	0.15
Sulfonamides	609 (0.4)	469 (0.2)	0.04
First-generation cephalosporins	1,890 (1.3)	4,201 (1.7)	0.03
Semi-synthetic penicillin	34,843 (23.2)	23,185 (9.1)	0.39

Note: number of SCr tests is presented in mean±SD; values expressed with number and a round bracket are presented as median [25th, 75th quartiles]; values expressed with number and a square bracket are presented as count (percentage).

Abbreviation: SCr, serum creatinine; AKI, acute kidney injury; CNY, Chinese Yuan; CCI, charlson comorbidity index; ICU, need for admission to the intensive care unit; CHD, coronary heart disease; CVD, cardiovascular disease; CKD, chronic kidney disease; CUSM, congenital urinary system malformation; SLE, systemic lupus erythematosus; GIB, gastrointestinal bleeding; COPD, chronic obstructive pulmonary disease; ACEI, angiotensin-converting enzyme inhibitor; ARB, angiotensin receptor blocker; NSAIDs, non-steroid anti-inflammatory drugs; PPI, proton pump inhibitor.

Table S3. The association between treatment with furosemide and risk of HA-AKI.

Subgroup	HA-AKI / N, (%)	Crude Model		Adjusted Model 1		Adjusted Model 2		Adjusted Model 3	
		HR (95% CI)	P value	HR (95% CI)	P value	HR (95% CI)	P value	HR (95% CI)	P value
Furosemide Versus Non-users									
Non-users	3,262 / 11,4815 (2.8)	Ref.		Ref.		Ref.		Ref.	
Furosemide	1,540 / 15,489 (9.9)	3.14 (3.03, 3.26)	<0.001	2.90 (2.80, 3.00)	<0.001	2.19 (2.11, 2.27)	<0.001	1.63 (1.57, 1.69)	<0.001
Furosemide Versus Other diuretics*									
Other diuretics	823 / 18,768 (4.4)	Ref.		Ref.		Ref.		Ref.	
Furosemide	1,540 / 15,489 (9.9)	2.40 (2.29, 2.51)	<0.001	2.28 (2.18, 2.39)	<0.001	1.70 (1.62, 1.79)	<0.001	1.33 (1.24, 1.42)	<0.001
Furosemide Versus Osmotic diuretics									
Osmotic diuretics	714 / 17,151 (4.2)	Ref.		Ref.		Ref.		Ref.	
Furosemide	1,540 / 15,489 (9.9)	2.17 (2.08, 2.28)	<0.001	1.99 (1.89, 2.08)	<0.001	1.54 (1.47, 1.61)	<0.001	1.11 (1.05, 1.18)	<0.001
Furosemide Versus Torasemide									
Torasemide	92 / 948 (9.7)	Ref.		Ref.		Ref.		Ref.	
Furosemide	1,540 / 15,489 (9.9)	1.63 (1.41, 1.88)	<0.001	1.60 (1.38, 1.84)	<0.001	1.65 (1.43, 1.91)	<0.001	1.33 (1.14, 1.54)	<0.001

Abbreviation: HA-AKI, hospital-acquired acute kidney injury; HR, hazard ratio; CI, confidence interval; Ref, reference.

*Other diuretics include thiazide, Low-ceiling (excluding thiazides), potassium-sparing and osmotic diuretics.

Model 1, adjusted for age, gender; Model 2, adjusted for estimated glomerular filtration rate (eGFR) and number of SCr tests, in addition to the variables in Model 1; Model 3, adjusted for the variables in Model 2, along with the Charlson comorbidity Index score, need for admission to the intensive care unit, mechanical ventilation, comorbidities, surgical procedures, use of other nephrotoxic drugs, and stratified by hospitals and divisions.

Table S4. The association between loop diuretics versus non-users and risk of HA-AKI in various subgroups.

Subgroup	Loop diuretics	Non-users	Adjusted HR* (95% CI)	P for interaction
	HA-AKI/ Total (%)	HA-AKI/ Total (%)		
**Age**				<0.001
18-40 year	192 / 2,332 (8.2)	551 / 27,001 (2.0)	1.54 (1.42, 1.68)	
40-65 year	815 / 8,815 (9.2)	1,803 / 62,635 (2.9)	1.59 (1.51, 1.67)	
65-100 year	625 / 5,290 (11.8)	908 / 25,179 (3.6)	1.75 (1.66, 1.85)	
**Gender**				<0.001
Male	1,089 / 10,284 (10.6)	2,051 / 58,406 (3.5)	1.54 (1.47, 1.61)	
Female	543 / 6,153 (8.8)	1,211 / 56,409 (2.1)	1.87 (1.76, 1.98)	
**Primary nephritis**				0.85
Yes	12 / 119 (10.1)	23 / 790 (2.9)	1.69 (1.13, 2.52)	
No	1,620 / 16,318 (9.9)	3,239 / 114,025 (2.8)	1.64 (1.58, 1.70)	
**Nephrotic syndrome**				0.58
Yes	46 / 367 (12.5)	68 / 1,309 (5.2)	1.73 (1.41, 2.12)	
No	1,586 / 16,070 (9.9)	3,194 /113,506 (2.8)	1.64 (1.57, 1.70)	
**Hypertension**				<0.001
Yes	99 / 941 (10.5)	220 / 6,562 (2.8)	1.23 (1.08, 1.40)	
No	1,533 / 15,496 (9.9)	3,042 / 108,253 (2.8)	1.67 (1.61, 1.74)	
**Heart failure**				0.004
Yes	87 / 352 (24.7)	53 / 507 (10.5)	1.28 (1.09, 1.52)	
No	1,545 / 16,085 (9.6)	3,209 / 114,308 (2.8)	1.65 (1.59, 1.72)	
**Liver cirrhosis**				0.003
Yes	50 / 693 (7.2)	83 / 1,439 (5.8)	1.22 (1.00, 1.50)	
No	1,582 / 15,744 (10.0)	3,179 / 113,376 (2.8)	1.65 (1.58, 1.71)	
**ICU**				0.66
Yes	242 / 1,404 (17.2)	268 / 5,216 (5.1)	1.66 (1.51, 1.83)	
No	1,490 / 15,033 (9.9)	2,994 / 109,599 (2.7)	1.63 (1.57, 1.70)	
**Operation**				<0.001
Yes	801 / 9,747 (8.2)	1,482 / 64,196 (2.3)	1.47 (1.38, 1.56)	
No	831 / 6,690 (12.4)	1,780 / 50,619 (3.5)	1.76 (1.68, 1.84)	
**ACEI/ARB**				0.56
Yes	200 / 1,756 (11.4)	312 / 10,130 (3.1)	1.58 (1.43, 1.75)	
No	1,432 / 14,681 (9.7)	2,950 / 104,685 (2.8)	1.65 (1.58, 1.71)	

Abbreviation: HA-AKI, hospital-acquired acute kidney injury; HR, hazard ratio; CI, confidence interval; ICU, need for admission to the intensive care unit; ACEI, angiotensin-converting enzyme inhibitor; ARB, angiotensin receptor blocker.

*adjusted for age, gender, estimated glomerular filtration rate, number of serum creatinine tests, Charlson comorbidity Index score, need for admission to the intensive care unit, mechanical ventilation, comorbidities, surgical procedures, use of other nephrotoxic drugs, and stratified by hospitals and divisions.

Table S5. The association between loop diuretics versus non-users and risk of HA-AKI in the 1:1 propensity score-matched cohorts (Method 1).

Subgroup	HA-AKI / N, (%)	Crude Model		Adjusted Model 1		Adjusted Model 2		Adjusted Model 3	
		HR (95% CI)	P value	HR (95% CI)	P value	HR (95% CI)	P value	HR (95% CI)	P value
Loop diuretics Matched Non-users									
Non-users	734 / 14,490 (5.1)	Ref.		Ref.		Ref.		Ref.	
Loop diuretics	1,375 / 14,490 (9.5)	1.75 (1.68, 1.83)	<0.001	1.13 (1.03, 1.23)	<0.001	1.73 (1.66, 1.81)	<0.001	1.52 (1.45, 1.59)	<0.001
Loop diuretics Matched Other diuretics*									
Other diuretics	266 / 3,655 (7.3)	Ref.		Ref.		Ref.		Ref.	
Loop diuretics	341 / 3,655 (9.3)	1.12 (1.03, 1.23)	0.009	1.13 (1.03, 1.23)	<0.001	1.11 (1.01, 1.21)	<0.001	1.21 (1.10, 1.34)	<0.001
Loop diuretics Matched Osmotic diuretics									
Osmotic diuretics	149 / 2,272 (6.6)	Ref.		Ref.		Ref.		Ref.	
Loop diuretics	208/ 2,272 (9.2)	1.28 (1.15, 1.41)	<0.001	1.26 (1.14, 1.40)	<0.001	1.18 (1.07, 1.31)	<0.001	1.14 (1.02, 1.27)	0.02

Abbreviation: HA-AKI, hospital-acquired acute kidney injury; HR, hazard ratio; CI, confidence interval; Ref, reference.

*Other diuretics include thiazide, Low-ceiling (excluding thiazides), potassium-sparing and osmotic diuretics.

Model 1, adjusted for age, gender; Model 2, adjusted for estimated glomerular filtration rate (eGFR) and number of SCr tests, in addition to the variables in Model 1; Model 3, adjusted for the variables in Model 2, along with the Charlson comorbidity Index score, need for admission to the intensive care unit, mechanical ventilation, comorbidities, surgical procedures, use of other nephrotoxic drugs, and stratified by hospitals and divisions.

Table S6. The association between loop diuretics versus other diuretics and risk of HA-AKI in the 1:1 propensity score-matched cohorts (Method 2).

Subgroup	HA-AKI / N, (%)	Crude Model		Adjusted Model 1		Adjusted Model 2		Adjusted Model 3	
		HR (95% CI)	P value	HR (95% CI)	P value	HR (95% CI)	P value	HR (95% CI)	P value
Loop diuretics Matched Non-users									
Non-users	754 / 14,686 (5.1)	Ref.		Ref.		Ref.		Ref.	
Loop diuretics	1,388 / 14,686 (9.5)	1.72 (1.65, 1.80)	<0.001	1.70 (1.63, 1.78)	<0.001	1.45 (1.39, 1.51)	<0.001	1.54 (1.47, 1.62)	<0.001
Loop diuretics Matched Other diuretics*									
Other diuretics	281 / 3,827 (7.3)	Ref.		Ref.		Ref.		Ref.	
Loop diuretics	394 / 3,827 (10.3)	1.39 (1.28, 1.51)	<0.001	1.38 (1.27, 1.50)	<0.001	1.29 (1.19, 1.40)	<0.001	1.23 (1.12, 1.35)	<0.001
Loop diuretics Matched Osmotic diuretics									
Osmotic diuretics	178 / 2,411 (7.4)	Ref.		Ref.		Ref.		Ref.	
Loop diuretics	246 / 2,411 (10.2)	1.55 (1.42, 1.69)	0.009	1.51 (1.38, 1.65)	<0.001	1.25 (1.15, 1.37)	<0.001	1.15 (1.04, 1.27)	<0.001

Abbreviation: HA-AKI, hospital-acquired acute kidney injury; HR, hazard ratio; CI, confidence interval; Ref, reference.

*Other diuretics include thiazide, Low-ceiling (excluding thiazides), potassium-sparing and osmotic diuretics.

Model 1, adjusted for age, gender; Model 2, adjusted for estimated glomerular filtration rate (eGFR) and number of SCr tests, in addition to the variables in Model 1; Model 3, adjusted for the variables in Model 2, along with the Charlson comorbidity Index score, need for admission to the intensive care unit, mechanical ventilation, comorbidities, surgical procedures, use of other nephrotoxic drugs, and stratified by hospitals and divisions.

Table S7. The association between loop diuretics versus osmotic diuretics and risk of HA-AKI in the 1:1 propensity score-matched cohorts (Method 3).

Subgroup	HA-AKI / N, (%)	Crude Model		Adjusted Model 1		Adjusted Model 2		Adjusted Model 3	
		HR (95% CI)	P value	HR (95% CI)	P value	HR (95% CI)	P value	HR (95% CI)	P value
Loop diuretic Matched Non-users									
Non-users	829 / 15,284 (5.4)	Ref.		Ref.		Ref.		Ref.	
Loop diuretics	1,494/ 15,284 (9.8)	1.78 (1.71, 1.86)	<0.001	1.76 (1.69, 1.84)	<0.001	1.49 (1.43, 1.55)	<0.001	1.53 (1.47, 1.60)	<0.001
Loop diuretics Matched Other diuretics*									
Other diuretics	295 / 3,992 (7.4)	Ref.		Ref.		Ref.		Ref.	
Loop diuretics	389 / 3,992 (9.7)	1.27 (1.17, 1.37)	<0.001	1.26 (1.16, 1.37)	<0.001	1.19 (1.10, 1.29)	<0.001	1.15 (1.05, 1.26)	0.002
Loop diuretics Matched Osmotic diuretics									
Osmotic diuretics	185 / 2,535 (7.3)	Ref.		Ref.		Ref.		Ref.	
Loop diuretics	276 / 2,535 (10.9)	1.43 (1.31, 1.56)	<0.001	1.39 (1.28, 1.52)	<0.001	1.22 (1.12, 1.33)	<0.001	1.15 (1.05, 1.27)	<0.001

Abbreviation: HA-AKI, hospital-acquired acute kidney injury; HR, hazard ratio; CI, confidence interval; Ref, reference.

*Other diuretics include thiazide, Low-ceiling (excluding thiazides), potassium-sparing and osmotic diuretics.

Model 1, adjusted for age, gender; Model 2, adjusted for estimated glomerular filtration rate (eGFR) and number of SCr tests, in addition to the variables in Model 1; Model 3, adjusted for the variables in Model 2, along with the Charlson comorbidity Index score, need for admission to the intensive care unit, mechanical ventilation, comorbidities, surgical procedures, use of other nephrotoxic drugs, and stratified by hospitals and divisions.

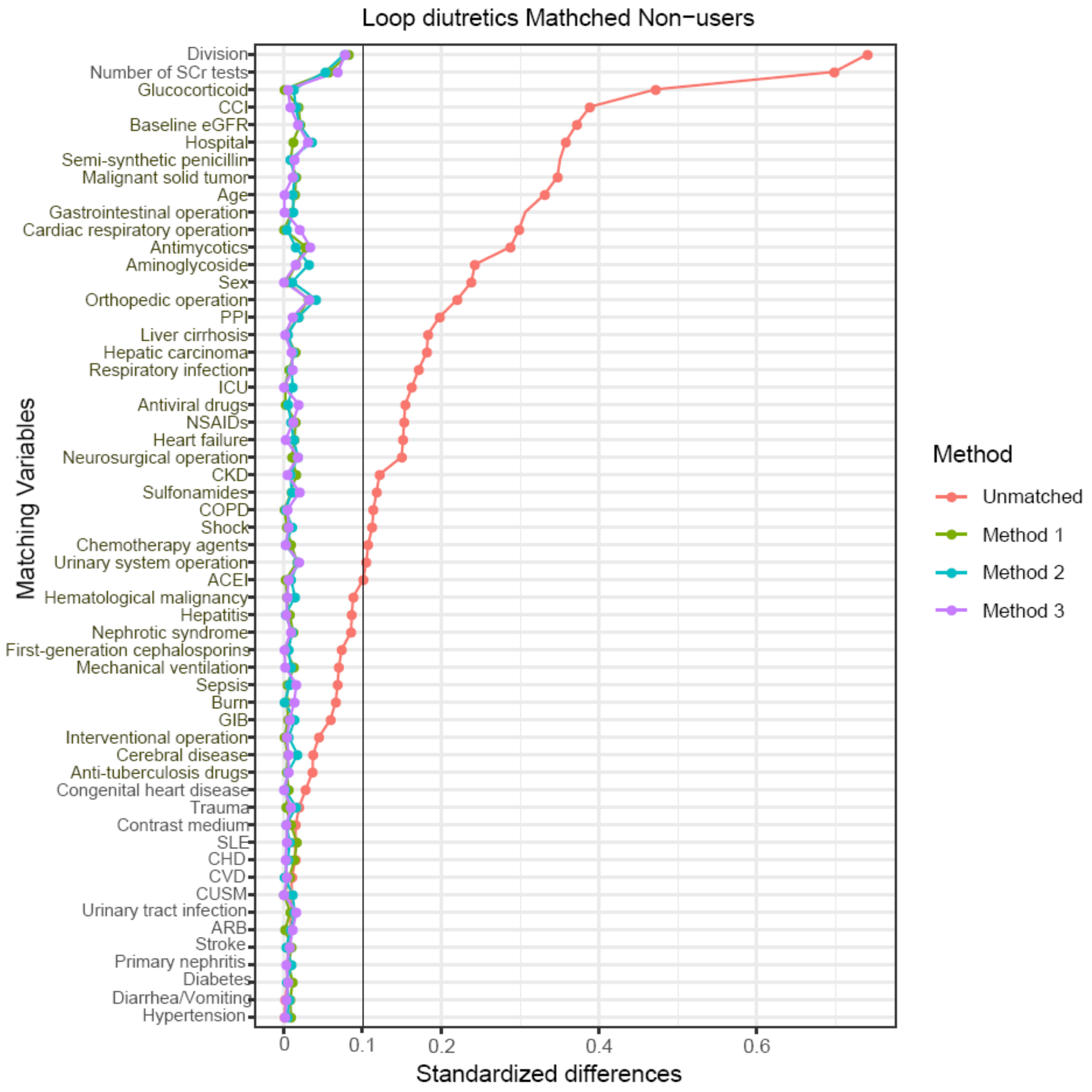

Figure S1. Distribution of standardized differences before and after 1:1 propensity score matching by three different models between loop diuretics and non-users. Method 1, nearest-neighbor matching without replacement and within a specified caliper width of 0.001; Method 2, nearest-neighbor matching without replacement and within a specified caliper width of 0.2 times the standard deviation of the logit of the estimated propensity score; Method 3, exact matching of age, sex, ICU, surgery, and nearest-neighbor matching without replacement and within a specified caliper width of 0.2 times the standard deviation of the logit of the estimated propensity score on other covariates. SCr, serum creatinine; CCI, Charlson comorbidity index score; eGFR, estimated glomerular filtration rate; PPI, proton pump inhibitor; ICU, need for admission to the intensive care unit; NSAIDs, non-steroid anti-inflammatory drugs; CKD, chronic kidney disease; COPD, chronic obstructive pulmonary disease; ACEI, angiotensin-converting enzyme inhibitor; GIB, gastrointestinal bleeding; SLE, systemic lupus erythematosus; CHD, coronary heart disease; CVD, cardiovascular disease; CUSM, congenital urinary system malformation; ARB, angiotensin receptor blocker.

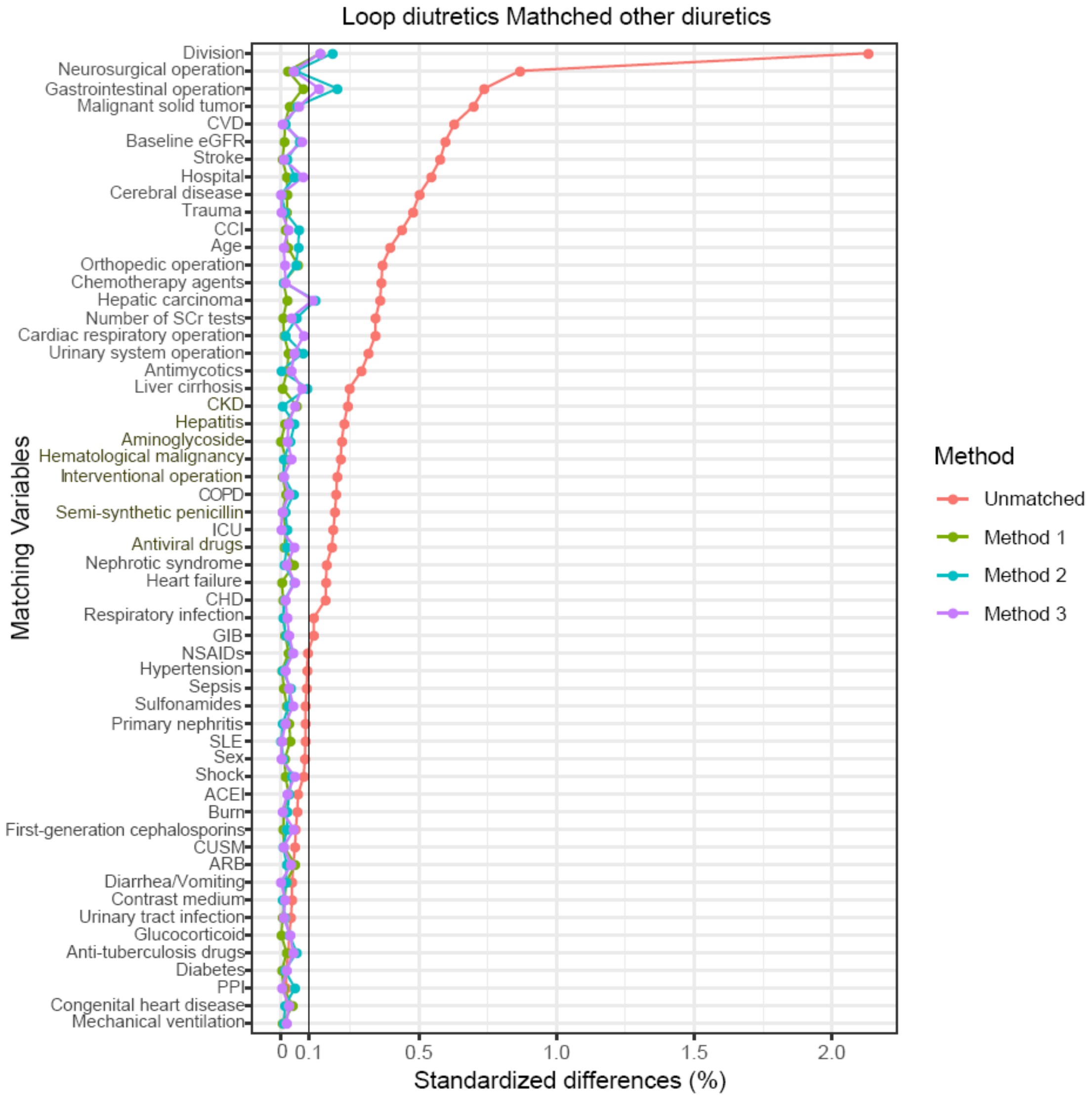

Figure S2. Distribution of standardized differences before and after 1:1 propensity score matching by three different models between loop diuretics and other diuretics. Method 1, nearest-neighbor matching without replacement and within a specified caliper width of 0.001; Method 2, nearest-neighbor matching without replacement and within a specified caliper width of 0.2 times the standard deviation of the logit of the estimated propensity score; Method 3, exact matching of age, sex, ICU, surgery, and nearest-neighbor matching without replacement and within a specified caliper width of 0.2 times the standard deviation of the logit of the estimated propensity score on other covariates. SCr, serum creatinine; CCI, Charlson comorbidity index score; eGFR, estimated glomerular filtration rate; PPI, proton pump inhibitor; ICU, need for admission to the intensive care unit; NSAIDs, non-steroid anti-inflammatory drugs; CKD, chronic kidney disease; COPD, chronic obstructive pulmonary disease; ACEI, angiotensin-converting enzyme inhibitor; GIB, gastrointestinal bleeding; SLE, systemic lupus erythematosus; CHD, coronary heart disease; CVD, cardiovascular disease; CUSM, congenital urinary system malformation; ARB, angiotensin receptor blocker.

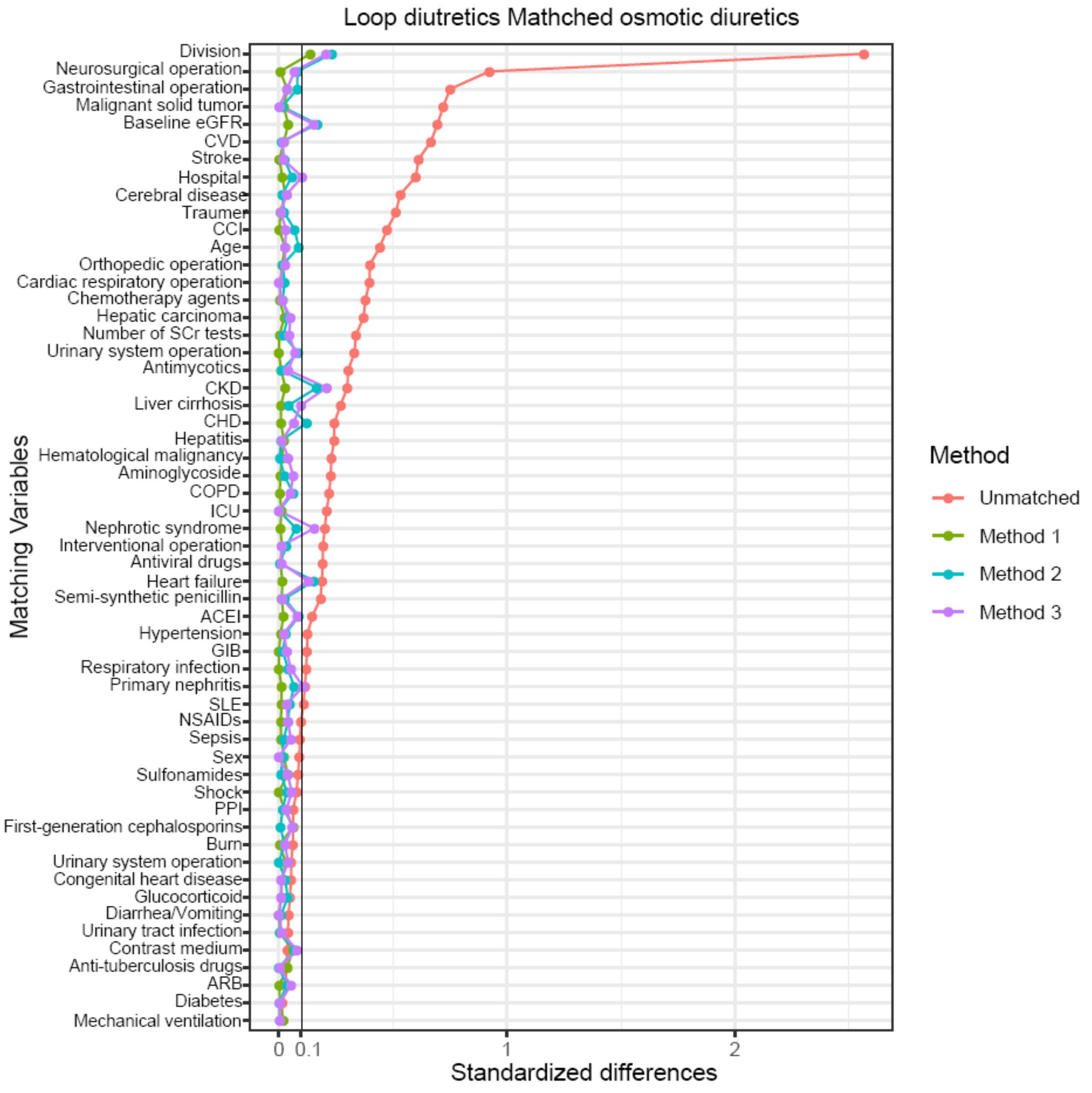

Figure S3. Distribution of standardized differences before and after 1:1 propensity score matching by three different models between loop diuretics and osmotic diuretics. Method 1, nearest-neighbor matching without replacement and within a specified caliper width of 0.001; Method 2, nearest-neighbor matching without replacement and within a specified caliper width of 0.2 times the standard deviation of the logit of the estimated propensity score; Method 3, exact matching of age, sex, ICU, surgery, and nearest-neighbor matching without replacement and within a specified caliper width of 0.2 times the standard deviation of the logit of the estimated propensity score on other covariates. SCr, serum creatinine; CCI, Charlson comorbidity index score; eGFR, estimated glomerular filtration rate; PPI, proton pump inhibitor; ICU, need for admission to the intensive care unit; NSAIDs, non-steroid anti-inflammatory drugs; CKD, chronic kidney disease; COPD, chronic obstructive pulmonary disease; ACEI, angiotensin-converting enzyme inhibitor; GIB, gastrointestinal bleeding; SLE, systemic lupus erythematosus; CHD, coronary heart disease; CVD, cardiovascular disease; CUSM, congenital urinary system malformation; ARB, angiotensin receptor blocker.
